# Identification of Novel Mutations in the *MMAA* and *MUT* Genes among Methylmalonic Aciduria Families

**DOI:** 10.61186/ibj.3782

**Published:** 2023-02-12

**Authors:** Mahboobeh Jafari, Fatemeh Karami, Aria Setoodeh, Ali Rahmanifar, Hamideh Bagherian, Mohammad Reza Alaei, Farzaneh Rohani, Sirous Zeinali

**Affiliations:** 1 Department of Biology, Science and Research Branch, Islamic Azad University, Tehran, Iran;; 2 Department of Medical Genetics, Applied Biophotonics Research Center, Science and Research Branch, Islamic Azad University, Tehran, Iran;; 3 Department of Pediatrics, Tehran University of Medical Sciences, Tehran, Iran;; 4 Clinical and Research Unit, Iranian National Society for the Study of Inborn Errors of Metabolism, Tehran, Iran;; 5 Kawsar Human Genetics Research Center, Tehran, Iran;; 6 Department of Pediatric Endocrinology and Metabolism, School of Medicine, Shahid Beheshti University of Medical Sciences, Tehran, Iran;; 7 Pediatric Growth and Development Research Center, Institute of Endocrinology and Metabolism, Iran University of Medical Science, Tehran, Iran;; 8 Department of Molecular Medicine, Biotechnology Research Center, Pasteur Institute of Iran, Tehran, Iran; # These authors were contributed equally in the present work.

**Keywords:** Autozygosity mapping, Genotype, Methylmalonic acidemia

## Abstract

**Background:**

Methylmalonic aciduria is a rare inherited metabolic disorder with autosomal recessive inheritance pattern. There are still MMA patients without known mutations in the responsible genes. This study aimed to identify mutations in Iranian MMA families using autozygosity mapping and NGS.

**Methods:**

Multiplex PCR was performed on DNAs isolated from 12 unrelated MMA patients and their family members using 19 STR markers flanking *MUT*, *MMAA*, and *MMAB* genes, followed by Sanger sequencing. WES was carried out in the patients with no mutation.

**Results:**

Haplotype analysis and Sanger sequencing revealed two novel, mutations, A252Vf*5 and G87R, within the *MMAA* and *MUT* genes, respectively. Three patients showed no mutations in either autozygosity mapping or NGS analysis.

**Conclusion:**

High-frequency mutations within exons 2 and 3 of *MUT* gene and exon 7 of *MMAB* gene are consistent with the global expected frequency of genetic variations among MMA patients.

## INTRODUCTION

Methylmalonic aciduria is a classic form of organic acidemia caused by mutations in genes encoding methylmalonyl‐CoA mutase and its co-enzyme, 5′-deoxyadenosylcobolamin. Prevalence of this disease varies worldwide and has recently been reported as 1.14 per 100,000 neonates^[^^1^^]^. According to reports of mutations disabling the critical genes, clinical presentations and onset of the MMA may vary among different patients and populations. MMA can be caused by the deficiency in either MUT enzyme or cobalamin (vitamin B12). Cobalamin deficiency occurs as the result of disturbances in the process of synthesis of adenosylcobalamin. MMA is also responsive to vitamin B12 supplement therapy and can be induced by mutations in *MMAA* (4q31), *MMAB* (12q24), and *MMADHC *genes or so-called cblA, cblB, and cblDv2 types, respectively. 

Current diagnosis of MMA is based on a combination of biochemical and genetic analyses. Genetic counseling for the suspected families can not only increase overall detection rate of the disease but also decrease the time and cost of unnecessary genetic tests^[^^2^^,^^3^^]^. Biochemical analysis includes detecting the level of urinary methylmalonic acid, 3-hydroxypropionate acid, citric acid, plasma glycine, valine, threonine, isoleucine, carnitine, and methionine. Genetic analysis often entails genotyping of *MMAA* (4q31), *MMAB* (12q24), and *MUT* (6p21) genes^[^^4^^]^. However, some of MMA patients have no known mutations in responsible genes (*MUT*,* MMAA*, and *MMAB*) in the patients with typical biochemical and clinical characteristics of MMA sometimes have no known mutation^[^^5^^]^. 

A recent study has reported five novel mutations, including c.805delG, c.693delC, c.223A>T, c.668A>G, and c.976A>G, within the *MUT* gene among Iranian patients, who are clinically diagnosed as MMA^[^^6^^]^. In another study, PCR sequencing analysis of *MMAA* gene in one family with two MMA siblings detected a homozygous deletion, c.674delA, in exon 4 of *MMAA *in both affected cases^[^^7^^]^. A separate study investigating three unrelated MMA patients by PCR sequencing reported one homozygous nucleotide change (c.2125-3 C>G) in the intron 12 of the *MUT* gene^[^^8^^]^. A research group found a novel C to G variation at the position -3 in the intron 12 of the *MUT* gene in two probands with definite diagnosis of MMA, which were associated with the retention of intron 12 in the final transcript product^[^^9^^]^. Herein, we performed autozygosity mapping using 19 STR markers flanking *MMAA*, *MMAB*, and *MUT* genes to identify candidate genes for MMA disease among the selected families. The identified genes were then subjected to PCR and Sanger sequencing and the negative cases were further analyzed by WES.

## MATERIALS AND METHODS

**Selection of patients**

Twelve unrelated Iranian patients with the same ethnicity, including six girls and six boys, affected by the isolated form of MMA were referred to Kawsar Human Genetics Research Center by pediatric endocrinologists. All the patients had a consanguineous marriage (first cousin). Diagnosis of the disease was based on the clinical presentations and the metabolite levels, methylmalonic acid and methylcitric acid. 

**Molecular genetic studies**

After genetic counseling for the patients’ families, 5 ml of whole blood was collected from all the cases and kept in EDTA-containing tubes. DNA was isolated from blood samples using the QIAamp DNA Mini Kit (Qiagen, Germany) and stored at -20°C until further steps. Finding STRs of the genes was performed using tandem repeat finder software. To consider the STR markers with higher heterozygosity, each marker was examined in 10 random samples. The designed primer pair sequences (using Primer3 software) with fluorescent primer binding site were employed for the amplification of STRs^[^^6^^]^. Selected STR markers were amplified in two independent multiplex PCR reaction, followed by fragment analysis by the ABI 3130 XL Genetic Analyzer (Thermo Fisher Scientific, USA). Haplotype maps were then separately drawn for all the families enrolled in the study with positive mutation. Cases with similar mutations in their family members were selected to be further analyzed by WES and carried out with targeted depth of 100×. Samples were prepared according to TruSeq Nano DNA library preparation and sequenced by an Illumina HiSeq X Ten plarform (Centogene, Rostok, Germany). After aligning the sequencing reads to the NCBI Build 38 of the human reference sequence and merging the alignments into a single BAM file, variants were defined through Genome Analysis Toolkit and then annotated using Ensemble Variant Effect Predictor. Positive cases, the only carriers of the mutation within their families, were subjected to further analysis by Sanger sequencing using specific primer pairs previously designed for all the exons of *MMAA*, *MMAB*, and *MUT* genes^[^^6^^]^. Sanger sequencing was performed on PCR products using a BigDye Terminator Kit (Thermo Fisher Scientific) according to the manufacturer’s protocol and then resolved on the ABI 3130xl genetic analyzer (Thermo Fisher Scientific) at the Kawsar Biotech Center facility. The identified novel mutations were genotyped by PCR Sanger sequencing in 100 Iranian healthy controls aged 17-60 years old, who were randomly referred to a cosmetic clinic to further determine their pathogenicity.

**In silico analysis**

The pathogenicity of the identified variants was investigated using different software, including Human Gene Mutation Database, MutationTaster, DynaMut, Fathmm, PolyPhen, and ClinVar.

## RESULTS

**Fig. 1:**
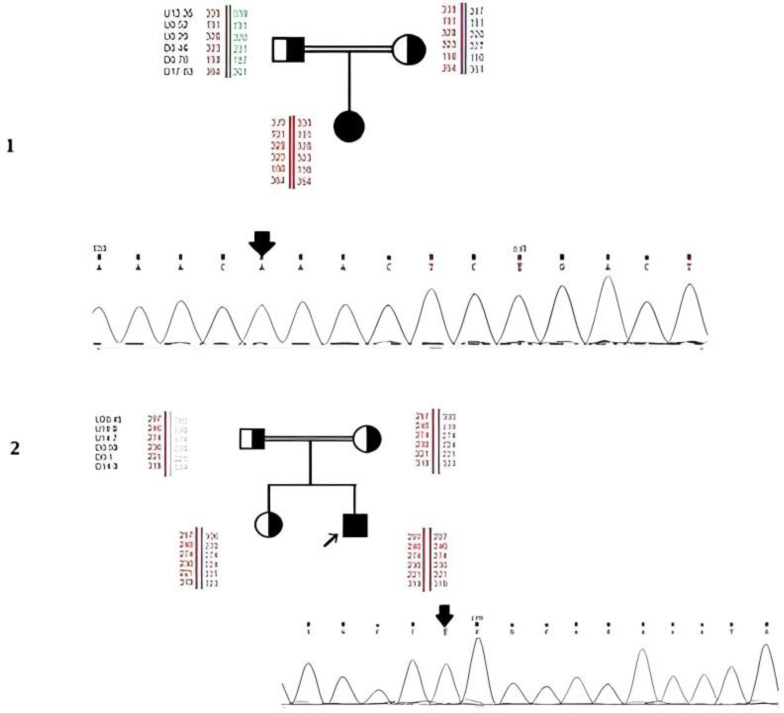
Haplotype analysis of the patient's families 8 and 3, along with Sanger sequencing confirmations. (1) Novel c.749-750ins (GTTT) insertion was identified in homozygote in an adult MMA patient using STR marker flanking *MMAA* gene. (2) Another novel c.259G>A mutation was found for the first time in an affected male MMA patient using STR marker flanking *MUT* gene

Autozygosity mapping and Sanger sequencing demonstrated homozygosity, including c.668A>G (K223R), c.259G>A (G87R), c.322C>T (R108C), c.1106G>A (R369H), and c.454C>T (R152X), for the *MUT* gene in five patients, while c.569G>A (R190H), IVS2-1G>T, and c.557G>A (R186Q) substitutions were found in other three patients in *MMAB* gene (Fig. 1). Moreover, c.749_750insGTTT (A252Vf*5) alteration was found in *MMAA* gene (Table 1; Supplementary Figs. 1 and 2). Three MMA patients revealed no homozygosity in none of the studied genes, and they were then subjected to further analysis by WES. In one of the patients examined by WES, c.380C>A (A127D) was found in the *MMAB *gene. In the second patient, c.628A>C (K210Q), c.901A>T (M301L), and c.1084A>T (M362L) were identified in the *ACSF3 *gene. No mutation was found in the third patient. The identified mutations were examined in family members by Sanger Sequencing to determine their role in the pathogenicity of MMA disease (Supplementary Fig. 3). The c.380C>A mutation was detected in homozygous status in the healthy brother of proband, and, therefore, its pathogenicity was ruled out. Owing to the presence of *ACSF3* gene mutations in other family members, the pathogenicity of those mutations was also ruled out (Supplementary Figure 3). First cousin of the latter case suffered from MMA. In the WES analysis, he demonstrated no mutation, including mutations in *ACSF3 *gene. Two other patients with homozygosity in the *MMAB* gene did not show any pathogenic mutations, and one patient showed homozygosity, c.749_750insGTTT, in the *MMAA* gene mutation. There are no previous reports on c.259G>A and c.749_750insGTTT mutations; therefore, they were introduced as novel cases. Parents of all the patients showed mutations in heterozygote form. There was no familial history among the enrolled families in terms of the inborn errors of metabolism, except for one family who had a male first cousin affected by MMA. He has previously been subjected to NGS analysis, and no mutation was found. It is worth to note that no novel mutation was identified in the control group.

**Table 1:** Genotype and phenotype characteristics of the patients analyzed by STR typing

**Gene**	**Exon**	**Mutation at** **protein level**	**Mutation at** **nucleotide level**	**Clinical symptoms at diagnosis**	**Current age (y)**	**Sex**	**Age at** **diagnosis**	**Patient** **No.**
*MUT*	2	R108C	c.322C>T	Severe developmental and growth delay	1	F	2 years	1
*MMAB*	7	R186Q	c.557G>A	Severe developmental and growth delay, Frequent vomiting and acidosis	2	M	1 years	2
*MUT*	2	G87R^*^	c.259G>A^*^	Developmental and growth delay, Frequent vomiting and acidosis	3	M	15 months	3
*MUT*	3	K223R	c.668A>G	Hypotonia, developmental delay	1	M	5 months	4
*MUT*	3	R152X	c.454C>T	Frequent vomiting and acidosis	2	F	10 months	5
*MMAB*	7	R190H	c.569G>A	Agitation, poor feeding	3	F	6 months	6
*MUT*	6	R369H	c.1106G>A	Poor feeding, hypotonia	7	M	3 days	7
*MMAA*	5	A252Vf*5^*^	749_750insGTTT^*^	Hypotonia	17	F	14 months	8
*MMAB*	2	-	IVS2-1G>T	Frequent vomiting and acidosis	16	F	2 years	9

*Novel mutation

## DISCUSSION

Herein, two novel mutations, c.259G>A (G87R) and c.749_750insGTTT insertion mutation, were found in the *MUT* and *MMAA* genes, respectively. Protein structure and function analysis of the c.259G>A mutation revealed that arginine substitution can cause deletion of 6 β-sheets, 32 α-helix, and B12-binding domain, which were associated with reduced protein stability. We also found a 15-month male patient with severe clinical presentations, including developmental delay, recurrent vomiting, and acidosis, which was compatible with the deleterious effect of mutation on protein function. Third novel mutation, 749_750insGTTT, was predicted to be associated with reduced enzyme activity, deletion of some peptides in alpha helix, and GTP-binding site of MMAA. This protein has a critical role in the exchange of co-factor of enzyme; thus, 749_750insGTTT mutation can impede recycling of coenzyme, which in turn decreases the enzyme activity^[10,11]^. Reduced enzyme activity with residual function is consistent with less severe and relatively late onset (14 months) presentation of our patient, including hypotonia as the major symptom in physical examination. Remaining mutations, i.e. c.569G>A, c.557G>A, and IVS2-1G>T in *MMAB* gene and c.1106G>A, c.454C>T, and c.322C>T in *MUT* gene have previously been reported in different studies. The c.569G>A substitution was found in a three-year-old female patient with major clinical presentations of poor feeding and agitation. Also, it has previously been reported in two separate studies and primarily found in two MMA type cblB patients with the early onset of the disease in both homozygote and heterozygote status^[^^11^^,^^12^^]^. Zhang et al.^[^^12^^]^ have demonstrated that histidine substitution at the 190 residue position can dramatically change the affinity of MMAB to adenosylcobalamin. Finding this mutation and displaying the clinical presentations in either homozygote or heterozygote status could be a further confirmation on the pathogenicity of this mutation. The c.557G>A mutation was the next recurrent mutation found as homozygote in a one-year-old male patient with severe developmental delay and acidosis. This mutation has formerly been reported in a white 14-year-old male patient with undefined clinical presentations, which has been predicted to affect enzyme activity through disrupting active site of protein and disabling its interaction with ATP in spite of normal enzyme production^[^^11^^]^. Furthermore, it has been found in two Canadian cblB type patients in both heterozygote and homozygote status^[^^13^^]^. While the c.557G>A mutation has been assigned as uncertain significance in ClinVar website, its replication in our patient with severe disease manifestations may clinically classify it as a definite pathogenic variant. 

The c.197-1G>T alteration was identified in a two-year-old female patient with vomiting and acidosis. It has also previously reported in two Arab patients and validated as pathogenic mutation^[^^14^^,^^15^^]^. The c.1106G>A mutation was determined in a three-day male neonate with poor feeding and lethargy. This mutation has been recognized as pathogenic variant and frequently reported in the patients with Caucasian origin in two earlier studies^[^^16^^,^^17^^]^. It was also characterized as catalytic mutation, which disrupts the highly conserved residue (arginine) at the position of 369. Additionally, the c.1106G>A substitution occurs in a CG-rich hot spot and it is always found as homozygote and associated with mut^0^ phenotype, which is in line with our findings^[^^16^^]^. The c.454C>T mutation was detected in our 10-month female patient with severe clinical presentations, including recurrent vomiting and acidosis. It is one of the most recurrent *MUT* gene mutations frequently reported in patients with the early onset severe clinical manifestations, which is in agreement with our patient's phenotype^[^^16^^,^^18^^-^^20^^]^. Data regarding the premature stop codon and truncated protein as the result of c.454C>T (R152X) mutation is also compatible with the early and severe disease demonstrations, which has been further replicated in the present study.

Prediction of the enzyme structure through online software demonstrated that c.668A>G substitution and replacement of lysine with arginine result in the reduced enzyme activity, as well as deletion of 26 alpha helix and vitamin B12-binding domain, which in turn disrupts the final protein structure and function. This mutation was found in a five-month male patient that his major clinical presentations were hypotonia, lethargy, and developmental delay, which are consistent with the severity of the identified mutation. The c.668A>G has previously been identified in a compound heterozygote Iranian MMA patient. Habibzadeh et al.^[^^21^^]^ have also found another truncating mutation (c.1055A>G) in exon 5 of MUT gene of a 15-month patient presenting the same clinical manifestations, as well as pneumonia aspiration.

All the identified mutations, except for one patient, were in homozygous status, which revealed as compound heterozygote, including c.628A>C, c.901A>T, and c.1084A>T. She was a 16-year-old patient who was absolutely normal till the 15 years of age and was then affected by sudden aphasia and hearing loss. Investigation of the mutations found within the *ACSF3 *gene in her parents indicated that they had no important defect in ACSF3 protein structure and function. Hence, severe late onset neurologic demonstrations might rely on the presence of possible mutation/s within *MMACHC* gene^[^^22^^]^.

One of the remarkable points of the present study is the clear genotype-phenotype correlation, which was found in most of the identified mutations, regardless of the patient number 2 with 557G>A mutation. Minimal thermal and stability changes in the protein structure, in spite of severe neurological manifestations, may indicate the presence of mutations in other responsible genes, including *MMACHC* and *MCEE*^[^^23^^]^.

Herein, we demonstrated that autozygosity mapping using selected STR markers can dramatically decrease the time and cost of the analysis for patients, particularly in those who were born from consanguineous marriage^[^^6^^]^. We could find pathogenic mutations in 9/12 (75%) of the patients. Regarding the patients with no mutations in the assayed exons, the most remained probability was mutations in other MMA responsible genes or cryptic mutations within the introns. There are limited reports on pathogenic intron mutations within three major *MUT*, *MMAA*, and *MMAB* genes, which were almost associated with splicing defect^[^^8^^,^^9^^,^^19^^]^. In addition, frequency of the mutations in the three main MMA genes has been estimated as 97%. Therefore, finding a mutation in two remaining genes may change the overall frequency of gene alterations at least in our population^[^^24^^]^. The frequency of the mutations was higher in exons 2 and 3 of the *MUT* gene and exon 7 of *MMAB* gene compared to other exons and responsible genes, which strongly corroborates most of the MMA genotyping studies performed previously^[^^11^^,^^17^^,^^25^^,^^26^^]^. 

Herein, two novel mutations, 749_750insGTTT and c.259G>A, were introduced within the *MMAA* and *MUT* genes, respectively. In silico analysis of the effects of the mutations on their corresponding protein structure and function was in agreement with clinical manifestations of the patients. However, we could not find any pathogenic mutations in the key MMA genes, in three patients. Further studies are warranted to determine the responsible mutations in other susceptible genes and the introns of *MUT*, *MMAA*, and *MMAB *genes to improve genetic counseling of patients with MMA disease.

## DISCUSSION

### Ethical statement

The study protocol was approved by the Ethics Committee of Kawsar Human Genetic Research Center, Tehran, Iran (ethical code: 98/6301). All the patients provided their written consents.

### Data availability

Data supporting this article are included within the article and supplementary file. 

### Author contributions

MJ: collected samples and performed laboratory assessment; FK: supervised the project, wrote the manuscript draft and performed the final manuscript revision; AS, AR, HB, MRA, FR: collected samples and analyzed the data; SZ: supervised the project and performed final manuscript revision.

### Conflict of interest

None declared.

### Funding/support

There is no funding for this project.

## Supplementary Materials


