# Supplementary material for: Identification of Novel Mutations in the MMAA and MUT Genes among Methylmalonic Aciduria Families
Source: Iran Biomed J. 2023 Feb 12;27(6):397–403. doi: 10.61186/ibj.3782 (PMC10826912; doi:10.61186/ibj.3782)
Supplement: Supplementary file 1 [file ibj-27-397-s001.pdf]

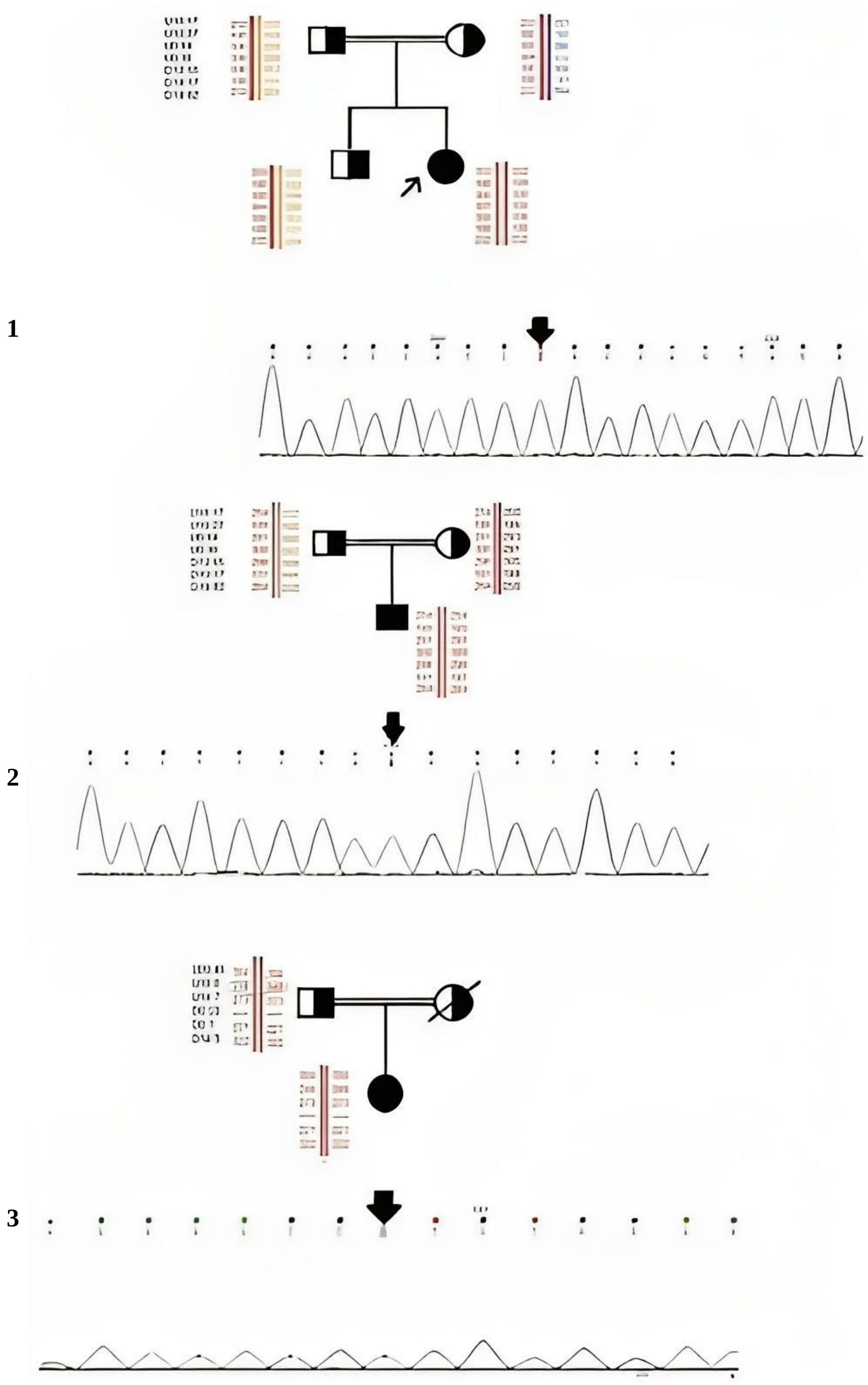

Supplementary Fig. 1. Haplotype analysis of the patient's families 1-3 along with Sanger sequencing confirmations. (1) Affected child was shown to be homozygote for c.322C>T mutation using STR marker flanking *MUT* gene. (2) A MMA boy with homozygote c.557G>A mutation using STR marker flanking *MMAB* gene. (3) A homozygote c.197-1G>T mutation was determined in a female adult patient within *MMAB* gene.

1

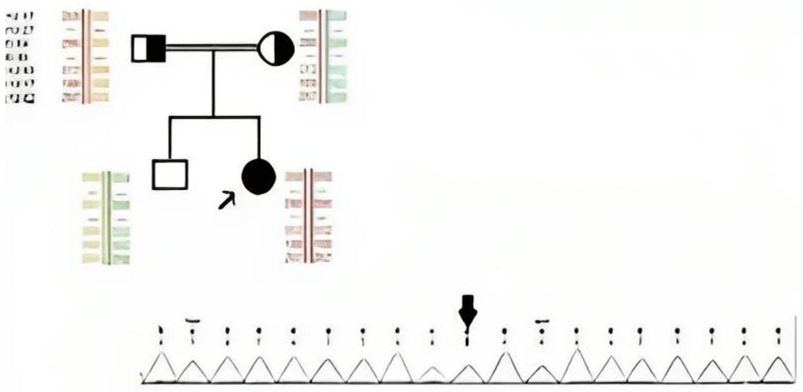

2

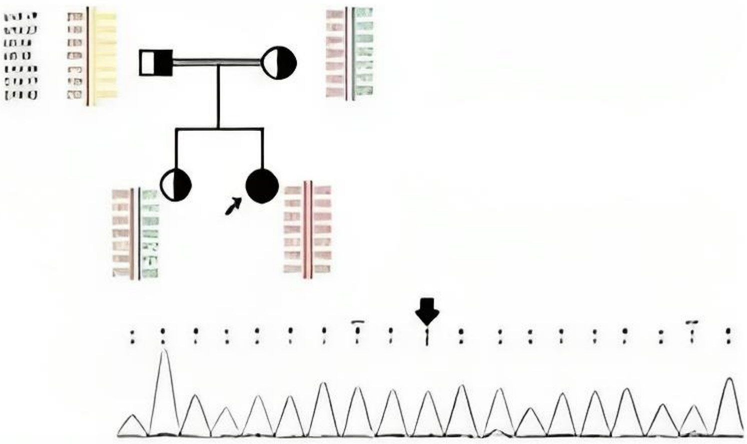

3

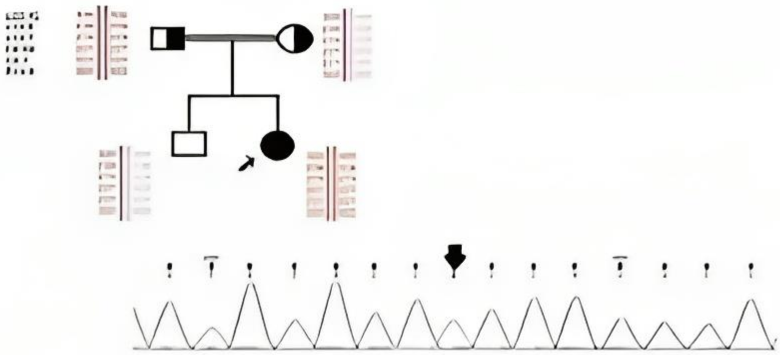

4

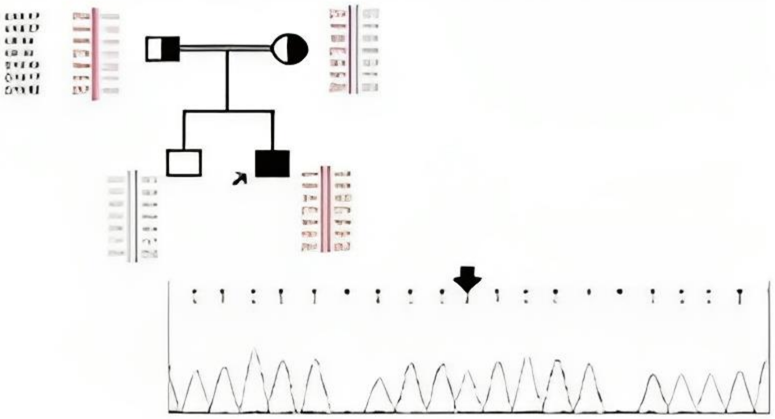

Supplementary Fig. 2. Haplotype analysis of the patient's families 4-7 along with Sanger sequencing confirmations. (1) It demonstrates an affected child with homozygote c.668A>G mutation in the *MUT* gene. (2) c.454C>T mutation was found in homozygote status in a female patient. (3) c.569G>A mutation was identified in homozygote form in a female patient using STR marker flanking *MMAB* gene. (4) c.1106G>A was found in homozygote status in a male patient with late onset demonstrations within *MUT* gene.

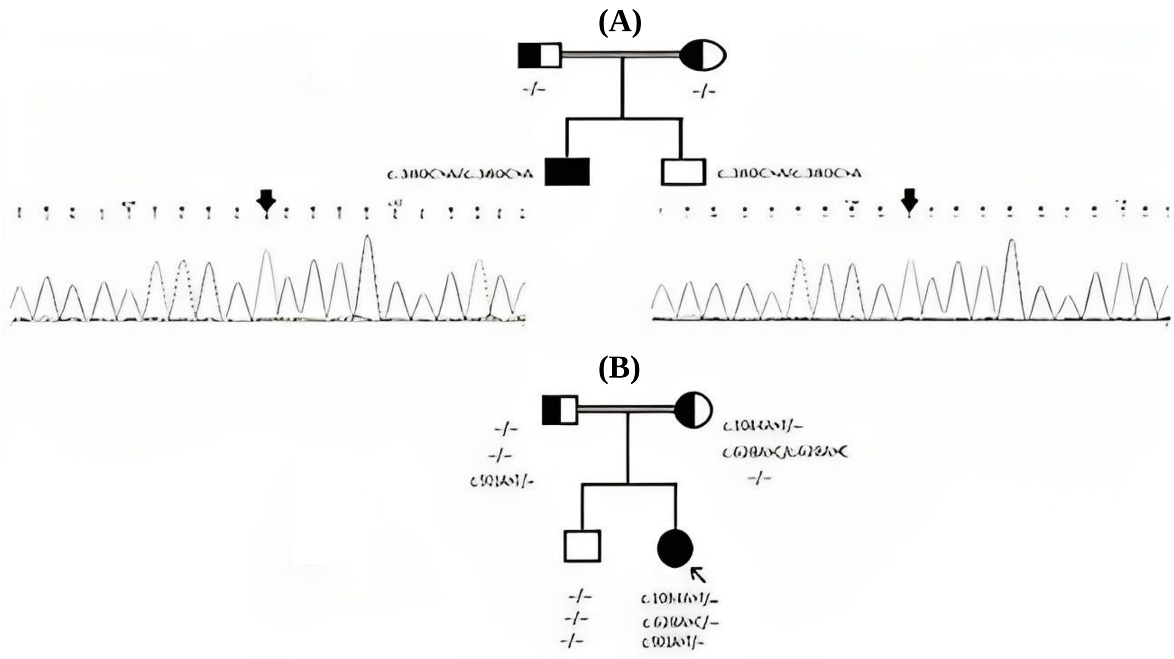

Supplementary Fig. 3. Pedigree and Sanger sequencing results of the patients analyzed by NGS. (A) Homozygote status of c.380C>A mutation in the patient and his healthy brother, indicating that it is not pathogenic; (B) a compound heterozygote patient that no one of them have important clinical effects according to the sequencing analysis of her parents.
